# Suicidal ideation among surgeons in Italy and Sweden – a cross-sectional study

**DOI:** 10.1186/s40359-014-0053-0

**Published:** 2014-11-29

**Authors:** Maja Wall, Karin Schenck-Gustafsson, Daria Minucci, Marie Gustafsson Sendén, Lise Tevik Løvseth, Ann Fridner

**Affiliations:** Department of Psychology, Stockholm University, Sweden, SE-10691 Sweden; Department of Medicine, Centre of Gender Medicine, Karolinska Institutet, Stockholm, Sweden; Departments of Obstetrics & Gynaecology, Padua University Hospital, Padua, Italy; Department of Research and Development, Division of Mental Health Care, St. Olav’s University Hospital, Trondheim, Norway

## Abstract

**Background:**

Suicidal ideation is more prevalent among physicians, compared to the population in general, but little is known about the factors behind surgeons’ suicidal ideation. A surgeon’s work environment can be competitive and characterised by degrading experiences, which could contribute to burnout, depression and even thoughts of suicide. Being a surgeon has been reported to be predictor for not seeking help when psychological distressed. The aim of the present study was to investigate to what extent surgeons in Italy and Sweden are affected by suicidal ideation, and how suicidal ideation can be associated with psychosocial work conditions.

**Methods:**

A cross-sectional study of surgeons was performed in Italy (N = 149) and Sweden (N = 272), where having suicidal ideation was the outcome variable. Work-related factors, such as harassment, depression and social support, were also measured.

**Results:**

Suicidal ideation within the previous twelve months was affirmatively reported by 18% of the Italian surgeons, and by 12% of the Swedish surgeons in the present study. The strongest association with having recent suicidal ideation for both countries was being subjected to degrading experiences/harassment at work by a senior physician. Sickness presenteeism, exhaustion and disengagement were related to recent suicidal ideation among Italian surgeons, while role conflicts and sickness presenteeism were associated with recent suicidal ideation in the Swedish group. For both countries, regular meetings to discuss situations at work were found to be protective.

**Conclusions:**

A high percentage of surgeons at two university hospitals in Italy and Sweden reported suicidal ideation during the year before the investigation. This reflects a tough workload, including sickness presenteeism, harassment at work, exhaustion/disengagement and role conflicts. Regular meetings to discuss work situations might be protective.

## Background

Suicidal ideation refers to a preoccupation with thoughts about suicide, and it is known to represent an early stage in the suicide process, from low mood and a passive death wish to specific plans to commit suicide, and finally self-harm (Casey et al. 2008). Having a suicide plan is associated with a significantly higher risk of carrying out a suicide attempt (Scocco et al. 2008). Suicide plans among physicians are often seriously intended, and much thought would have been put into the self-destruction factors; they may even have rehearsed how to do it (Myers 2011). In Europe, suicidal ideation varies in the general population between countries, with prevalence between 2-14% (Bernal et al. 2007). In Sweden, the prevalence of suicidal thoughts during the last twelve months is reported to be about seven per cent in general (Ramberg and Wasserman 2000), while lifetime prevalence in Italy has been reported to be three per cent, and found to be more common among female Italians (Scocco et al. 2008).

Research has found suicidal ideation to be highly prevalent among physicians, and they are at high risk of suicide (Bertges Yost et al. 2005; Fridner et al. 2009, 2011). Approximately 13-14% of Swedish academic physicians report that they have had suicidal thoughts within the previous 12 months (Fridner et al. 2011). When controlling for socio-economic and demographic factors, physicians are the only profession with an increasing level of suicidal thoughts (Agerbro et al. 2007). Physicians may be as much as 2.45 times more likely to commit suicide than non-physicians. Emotional distress, responsibility for patients’ problems, and inter-role conflict may lead to feelings of bearing a burden (Cornette et al. 2009). There needs to be focus on psychosocial work conditions among physicians, as they in addition, are assumed to be at increased risk of burnout and job dissatisfaction (Robstad Andersen et al. 2010). To prevent suicide among physicians, it is essential to identify potential risk for, and protective factors against, suicidal ideation.

Among anaesthesiologists, 25% suffer from suicidal ideation, and one contributing factor has been found to be conflicts with colleagues or superiors (Lindfors et al. 2009). Among Swedish physicians, 14 percent have reported themselves to be victims of harassment during the previous six months. In addition, 42 percent reported that the perpetrators were superiors and 49 percent reported the perpetrators to be colleagues (Robstad Andersen et al. 2010). When asking Italian physicians, more than 20 percent had been subjected to harassment, where the perpetrators in 76 percent of the cases were superiors, and in approximately 40 percent of the cases, colleagues (Robstad Andersen et al. 2010). Among hospital personnel, being subjected to harassment once a week during the previous six months, was reported by 19% (Fornés et al. 2011). Being psychologically harassed has been found to cause stress and a drop in self-confidence, and this could additionally affect one’s health (Fornés et al. 2011). Accordingly, harassment at work can affect health care personnel, and may in the long run contribute to burnout and distress.

According to Campbell et al. (Campbell et al. 2001) over 30 percent of American surgeons suffer from burnout, and in addition, studies on Dutch physicians have reported that over 20 percent are classified as suffering burnout (Campbell et al. 2001; van der Heijden et al. 2008). Burnout, which refers to a person’s relationship with work, may lead to depersonalisation, where the surgeon fails to view the patient as a human being, which may affect the ability to provide good patient care (Campbell et al. 2001). Burnout has been found to affect younger ages, according to a study among American oncology surgeons, and as surgeon burnout may concern themselves, families, patients and colleagues, it is likely to affect the surgeon both personally and professionally (Fornés et al. 2011; Balch and Shanafelt 2011).

As mentioned above, symptoms of depression have in general been associated with suicidal ideation, and 43 percent of physicians working in academic medical settings suffer from depression or burnout (Bernal et al. 2007; Lindfors et al. 2009; Fridner et al. 2012). Being a surgeon has in addition been found to be a predictor of not seeking help when suffering from psychological distress (Fridner et al. 2012). Being depressed could be a result of not being able to control one’s own environment, and when surgeons experience high demands at work and a lack of efficiency and autonomy, they might feel that they are not in control of their work performance (Klein et al. 2011; Sharot 2011). Depressive symptoms could also be fostered by work-family conflicts, where one might experience an imbalance between work and family life. Such imbalance has been found to contribute to distress among female surgeons (Cornette et al. 2009; Dyrbye et al. 2011). This result has been supported by a study on veterinary surgeons, which reported that the imbalance between work and family may contribute to poor mental well-being (Platt et al. 2012).

The academic clinical setting can be a challenging and demanding work environment involving medical research, clinical work and teaching. To attain the profession of surgeons, medical trainees have to undergo years of study and practice, with new demands replacing previous challenges (Balch and Shanafelt 2010; Shanafelt 2008). They maintain a strategy of delaying gratification, which may affect their personal well-being and lead to burnout (Shanafelt et al. 2011). Surgeons with fewer years in practice tend to feel that work is overwhelming and that working as a surgeon does not meet their expectations, especially in the competitive working environment of academic hospitals (Campbell et al. 2001). A highly competitive environment could foster sickness presenteeism, where one prefers to go to work ill than stay at home to recover (Fridner et al. 2009; Gustafsson Sendén et al. 2013). By attending work despite illness, or taking compensatory leave, one covers the weaknesses that may be perceived as negative in a competitive workplace (Fridner 2004). Sickness presenteeism has been found to be associated with role conflicts, which in turn are likely mediators for mental illness and are associated with suicidal ideation (Gustafsson Sendén et al. 2013; Haines et al. 2008).

Earlier studies have reported that regular meetings to discuss stressful situations at work contribute to a better working environment, and in the long run, reduce the risk of suicidal ideation (Fridner et al. 2011; Wada et al. 2011). Additionally, the meetings can provide clearer information about roles and work situations (Robstad Andersen et al. 2010).

### Aim of the study

As one of the first European cross-sectional studies on suicidal ideation among surgeons, our aim is to investigate to what extent surgeons in Italy and Sweden are affected by suicidal ideation. In addition, we want to compare the work-related factors associated with suicidal ideation in Italy and Sweden more closely, especially since we previously have found surgeon to be predictor for psychological distress (Fridner et al. 2012). What factors in the surgeons’ work place expose them to higher risk of distress? On the basis of recent research on suicidal ideation in the general population in respective country, we expect the rate of suicidal ideation to be lower among Italian surgeons than among Swedish surgeons.

We hypothesise that harassment can contribute to surgeons’ suicidal ideation. Burnout, role conflicts and sickness presenteeism can also lead to suicidal ideation, while regular meetings to discuss stressful situations at work may be a protective factor against suicidal ideation among Italian and Swedish surgeons.

## Methods

### Settings

This is a cross-sectional study, based on data from the first phase of the Health and Organization among University hospital Physicians in Europe (HOUPE) study. For the purpose of the present study the subjects chosen were in specific surgeons working in Italy and Sweden.

In Italy, the study took place at Padua University Hospital, and was approved by The Ethics Board of Padua University Hospital, on September 5, 2005, protocol number 1039P. All eligible Italian physicians received a letter shortly thereafter describing the study. The Swedish study was carried out at Karolinska University Hospital, with approval from the Regional Ethics Board (Stockholm), on December 8, 2004, protocol number 04-913/2. The data collection was organised through a web-based survey. All physicians received written information about the survey, often supplemented by short oral presentations. Four reminders were sent via e-mail, and a paper version was also sent to all of the physicians. The participants used personal login information to enter their responses to the web-based survey anonymously. In Sweden, the questionnaire was written in English, while the Italian version was validated by back translation between English and Italian. The questionnaire contained 116 questions concerning work-related health, organisational culture and working conditions.

### Participants

Physicians were eligible to participate if they were permanently employed as physicians and actively working at the respective university hospitals during the data collection period. All physicians were invited to participate in the study on a voluntary basis, with confidentiality guaranteed, and it was emphasized that no individual data could be identified in any way.

Altogether, 1380 Swedish physicians and 900 Italian physicians were invited to participate in the HOUPE study (phase 1). The response rate was 59.8% in Sweden and 41.3% in Italy, and the response rate for female physicians was slightly higher than for male physicians (56.2% and 47.6% respectively). The respondents comprised 272 Swedish surgeons and 149 Italian surgeons.

### Dependent variable

The outcome variable “recent suicidal ideation” was assessed via dichotomous (yes/no) questions concerning the past 12 months. The queries were: “Have you had thoughts about taking your life?” and “Have you had thoughts about specific ways to take your life?” (Meehan et al. 1992). We combined the two queries to create a composite dichotomous dependent variable. An affirmative response to either of the questions was considered to indicate the occurrence of recent suicidal thoughts.

### Independent variables

Psychological distress was measured with the 12-item version of the General Health Questionnaire (GHQ-12) (α =0.893, e.g. “Have you recently lost much sleep over worry?”) Answers ranged from 1 (Never) to 4 (Always). (Goldberg and Williams 1991). The “Mini Oldenburg Burnout Inventory” MOLBI assessed burnout via two additive scales, with five items each for disengagement (α = 0.762, e.g. “I usually talk about my work in a derogatory way”) and for exhaustion (α = 0.792, e.g. “During my work, I often feel emotionally drained”). Answers ranged from 1 (Totally agree) to 4 (Totally disagree) (Demerouti et al. 2003). All the queries from the GHQ-12 and MOLBI referred explicitly to the past few weeks.

The Questionnaire for Psychological and Social Factors at Work (QPSNordic) (Lindström 2000) was used to assess recent degrading experiences or harassment at work and work-related factors. The question for measuring *harassment or degrading experiences at work* was “Have you been subjected to harassment at your workplace during the recent six months?” with answers “Yes” or “No”. It was introduced by the following definition: “Harassment and degrading experiences are a problem at some workplaces and for some employees. To label something as harassment, the offensive behaviour has to occur repeatedly over a period of time, where the subject feel incapable of defending oneself”. Then followed a question regarding the perpetrator of the harassment, in which respondents were asked to specify by whom they had been harassed (i.e. a colleague, a superior, patients).

For measuring *role conflict* a three-item scale was used (α = 0.721, e.g. “Do you have to do things that you feel should be done differently?), with answers ranging from 1 (very seldom or never) to 5 (very often or always). *Empowering leadership* was measured according to a three-item scale (α = 0.872, e.g. “Does your immediate superior encourage you to participate in important decisions?”). The responses were answered on a five-point scale ranging from 1 (very seldom or never) to 5 (very often or always). *Predictability at work* was assessed with a single item (“Do you feel you have someone or an organisation which looks after your interests?”) and measured on a five-point scale from 1 (very seldom or never) to 5 (very often or always). The *work-home interface* was assessed by a two-item scale (α = 0.548, “Do the demands of work interfere with family life?” and “Do the demands of family or spouse/partner interfere with work-related activities?”). The responses were measured on a five-point scale from 1 (very seldom or never) to 5 (very often or always). The QPSNordic questionnaire was recently tested for validity and reliability by test-retest measures and by collecting data from various branches (Lindström 2000).

One single-item question was “*Are there regular meetings to discuss stressful situations at work?” and was* conducted from a Norwegian research program concerning physicians’ health (Aasland and Falkum 1994). Answers ranged from one to three (No; It happens from time to time, but nothing formal; Yes). There was also a question concerning *hours on night-call* given. One item was assessed to measure whether participants *went to work with an illness* for which they would recommend a patient to stay at home (Rosvold and Bjertness 2002), and one item from the Physician Career Path Questionnaire (PCPQ) (Fridner 2004) was assessed to measure if they had *taken compensatory leave or holiday due to stress.* Both items were measured on a five-point scale ranging from 1 (very seldom or never) to 5 (very often or always). In addition to gender and country, items concerning age, civil status (married/living with a partner versus living alone (single, divorced or widowed), and number of children were presented.

### Statistical analysis

Characteristics such as age group, gender, civil status, and number of children of the population were defined by numerical count and percentages. Data analyses included comparisons between Swedish and Italian surgeons using Chi-square tests for categorical/semi-continuous variables, and using two-sample *t*-tests for comparing means of the groups. Bivariate logistic regressions were conducted to identify work-related factors and health-related behaviours or confounders with significant odds ratio for suicidal ideation. The multivariate logistic regression was done separately for the Italian and Swedish surgeons, with a model computed for each country, in which all variables were put together. All analyses were carried out using the statistical software SPSS 21.

## Results

There were significantly more female Swedish surgeons (39%) than female Italian surgeons (24%). However, when comparing the frequencies of suicidal ideation among female and male surgeons, no differences were found in the results (χ^2^_1_ = 0.438, *ns*). There were no differences in age (χ^2^_2_ = 0.690, *ns*) or marital status (χ^2^_1_ = 3.058, *ns*) between Italian and Swedish surgeons.

Twelve percent of the Swedish surgeons and 18% of the Italian affirmatively reported suicidal ideation within the last twelve months. Table 1 shows descriptive data stratified to compare the characteristics of the surgeons in Sweden and Italy. Significantly more Italian physicians were male, did not have any children, had less night-call duty and less support from their organisation, and specifically from their leaders. They had significantly often experienced recent degrading experiences/harassment from their senior physician compared to the Swedish surgeons. In Table 2 we report, by using bivariate logistic regression, the statistically significant association between the independent non-adjusted variables and the outcome variable. The strongest bivariate association with having recent suicidal ideation for both Italian and Swedish surgeons was being harassed by a senior physician (OR, 3.26; 95% CI, 1.34-7.92 and OR, 5.83; CI% 2.38-14.28, respectively). However, hours on night call, compensatory leave and empowering leadership were not associated with suicidal ideation.

**Table 1:** **Comparison of demographic characteristics, work-related factors, behavioural factors and psychological distress for Swedish and Italian surgeons**

**Characteristics**		**Sweden (%)**	**Italy (%)**	p*
**Number of respondents**		**272**	**149**	
Gender	Male	166 (61.0)	113 (75.8)	
	Female	106 (39.0)	36 (24.2)	.002
	Missing data	0	0	
Age	<40 y	28 (10.3)	18 (12.2)	ns
	40- < 54 y	170 (62.7)	87 (58.8)	
	≥55 y	73 (26.9)	43 (29.1)	
	Missing data	0	0	
Living with partner	Yes	235 (88.8)	121 (81.2)	ns
	No	37 (11.2)	28 (18.8)	
	Missing data	0	0	
Number of children	0	39 (14.6)	40 (27.2)	.000
	1-2	131 (49.1)	86 (58.5)	
	3 or more	97 (36.3)	21 (14.3)	
	Missing data	5	2	
**Work-related factors**				
Recent degrading experiences/harassment at work	Yes	49 (18.0)	45 (30.2)	ns
	No	212 (77.9)	102 (68.5)	
	Missing data	11	2	
Perpetrator, superior	Yes	27 (9.9)	38 (25.5)	.000
	No	245 (90.1)	111 (74.5)	
	Missing data	0	0	
Perpetrator, colleague	Yes	23 (8.5)	17 (11.4)	ns
	No	249 (91.5)	132 (88.6)	
	Missing data	0	0	
Hours on night call, mean (SD)		8.82 (13.1)	5.93 (4.57)	.028
Taken care of in the organisation, mean (SD)		2.49 (1.03)	1.60 (0.93)	.000
Regular meetings to discuss situations at work, mean (SD)		2.92 (0.88)	2.66 (0.90)	.005
Role conflict, mean (SD)		2.93 (0.88)	3.04 (0.95)	ns
Empowering leadership, mean (SD)		2.92 (1.13)	2.57 (1.23)	.004
**Work/home interface and behavioural factors**				
Work demands interfere with family life, mean (SD)		3.71 (0.95)	3.77 (1.01)	ns
Family life interferes with work, mean (SD)		2.66 (1.13)	2.50 (1.03)	ns
Sickness presenteeism, mean (SD)		3.19 (1.16)	3.34 (1.09)	ns
Compensatory leave, mean (SD)		1.33 (0.77)	1.25 (0.68)	ns
**Psychological distress**				
Suicidal ideation in last 12 months, number (%)	Yes	32 (11.8)	26 (17.4)	ns
	No	235 (86.4)	120 (80.5)	
	Missing data	5	3	
Depression, mean (SD)		2.09 (0.47)	2.01 (0.44)	ns
Burnout exhaustion, mean (SD)		2.57 (0.51)	2.49 (0.58)	ns
Burnout disengagement, mean (SD)		2.28 (0.52)	2.17 (0.57)	ns

*p-values measured with χ^2^ tests (−1 df) for dichotomous variables, t-test comparing means. Differences in scale scores assessed by 2-sample t-tests, 2-tailed significant levels.

**Table 2:** **Factors with significant (** ***p*** **≤0.05) non-adjusted odds ratios (ORs) for recent suicidal thoughts among Italian and Swedish surgeons**

**Group**	**Independent variables**	**OR**	**95% CI**	**p**
**Sweden**				
Work-related	Recent degrading experiences/harassment at work	4.10	1.83 – 9.17	0.001
	Role conflict	1.85	1.17 – 2.91	0.008
	Regular meetings to discuss situations at work	0.49	0.28 - 0.85	0.012
Work-family interface & health-related behaviours	Work demands interfere with family life	1.59	1.03 – 2.48	0.038
	Sickness presenteeism	1.63	1.14 – 2.33	0.008
Psychological distress	Disengagement	3.85	1.86 – 7.97	0.001
**Italy**				
Work-related	Recent degrading experiences/harassment at work	2.97	1.24 – 7.10	0.015
	Recent degrading experiences/harassment at work by superior	3.26	1.34 – 7.92	0.009
Work-family interface & health-related behaviours	Sickness presenteeism	1.68	1.08 – 2.60	0.021
Psychological distress	Exhaustion	2.71	1.22 – 6.01	0.014
	Disengagement	2.50	1.19 – 5.25	0.015

Bivariate logistic regression.

For the Swedish surgeons, harassment or degrading experiences at work were associated with recent suicidal ideation (OR, 2.83; 95% CI 1.13-7.09). Disengagement at work was the only psychological distress variable associated with the outcome variable among the Swedish surgeons (OR, 2.95; 95% CI 1.26-6.94). In addition, having regular meetings to discuss work was protectively associated with having suicidal thoughts among the Swedish surgeons (OR, 0.51; 95% CI 0.27-0.98). The results for the Italian surgeons showed that sickness presenteeism (OR, 1.74; 95% CI 1.11-2.74) and disengagement (OR, 2.96; 95% CI 1.32-6.64) were related to recent suicidal ideation (see Table 3).

**Table 3:** **Factors with significant (p ≤0.05) adjusted odds ratios (ORs) for recent suicidal thoughts among Italian and Swedish surgeons**

**Group**	**Independent variable**	**Adjusted OR**	**95% CI**
**Sweden**	Disengagement	2.95	1.26-6.94
	Recent degrading experience/harassment at work	2.83	1.13-7.09
	Regular meetings to discuss experiences at work	0.51	0.27-0.98
**Italy**	Disengagement	2.96	1.32-6.64
	Sickness presenteeism	1.74	1.11-2.74

Multivariate regression with adjustment for non-significant socio-demographic factors; age, number of children and marital status.

## Discussion

The focus of this study was to examine the associations between work stressors and suicidal ideation among Swedish and Italian academic medical surgeons. The aim was to investigate to what extent surgeons in Italy and Sweden are affected by suicidal ideation and, in addition, to what extent suicidal ideation is associated with work-related factors.

Our findings indicate that surgeons’ suicidal ideation is associated with a poor work environment, including harassment and sickness presenteeism, but also that regular meetings to discuss situations at work could be protective. One important finding was that 18 percent of the Italian surgeons, and 12 percent of the Swedish surgeons, reported suffering from suicidal ideation during the previous 12 months. The result for the Swedish surgeons is higher than previously found among physicians in general (8,70-9,70%), and also higher than previous findings on surgeons (6.5%) (Fridner et al. 2009, 2011; Dyrbye et al. 2011). However, the fact that almost one of five of our Italian surgeons was found to have suicidal ideation is noteable, since it is almost twice as many as Italian physicians in general (8,75-10,30%) (Fridner et al. 2009, 2011). In contrast to other studies, we found no gender differences in suicidal ideation between female and male surgeons in our analyses.

One key finding is that in the bivariate logistic regression, harassment was the strongest associated predictor for suicidal ideation. However, in the multivariate model, harassment was associated with suicidal ideation only for the Swedish surgeons. This means that this is one of the work environmental factors associated with suicidal ideation. This is worrying because suicidal ideation has been found to be a precursor to succeeding in suicide, and also because physicians have knowledge of how to succeed in suicide (Robstad Andersen et al. 2010). Concerning harassment, we found that a large percentage of the surgeons had recently experienced harassment at work, from colleagues as well as superiors. Almost one-third of the Italian surgeons (30,2%) and one in five Swedish surgeons (18%) had experienced harassment or degrading experiences. This is high compared to reported harassment frequency in the general population in Sweden (3.5-9%) and two percent in Italy (Robstad Andersen et al. 2010). Harassment from patients and their families has previously been reported to contribute to physicians’ suicidal ideation (Wada et al. 2011); however, no such result was found among the surgeons in the present study. Additionally, no gender differences in experiencing harassment were found. This contradicts previous findings that female surgeons more frequently report being subjected to humiliation and harassment (Wallace 2013). The present study may contribute new knowledge that male surgeons also experience harassment at work.

In contradiction to previous findings, no association was found between depression and suicidal ideation. One explanation may be that we used the validated GHQ-12 scale, in contrast to the other study, where only a single item was used (Dyrbye et al. 2011). However, as previously found, suicidal ideation may not necessarily be preceded by major depression (Rhodes and Bethell 2008). On the contrary, we found that the strongest associated predictor for suicide ideation was disengagement, which is one of the two dimensions of burnout. Our findings are in line with previous studies, which have reported surgeon burnout to be higher than in the general population (Bertges Yost et al. 2005; Fornés et al. 2011; Balch and Shanafelt 2011). Being disengaged from one’s work could lead to a risk of depersonalizing patients and their relatives. Furthermore, this may affect surgeons’ work quality and patient care (Campbell et al. 2001). In addition, we now suggest that disengagement may indicate suicidal ideation. On this basis, it is important to spread this knowledge to management and HR, so that they can offer better institutional support to their employees.

Sickness presenteeism was associated with suicidal ideation among both Italian and Swedish surgeons. The findings have suggested that surgeons feel they cannot miss out on work or stay at home when sick, due to a competitive work environment, low compensation and the effort one has to put in as a surgeon (Shanafelt 2008; Gustafsson Sendén et al. 2013). Such behaviour, of working while sick, might also affect their work efficacy and the quality of patient care, while the risk of medical errors might increase (Wada et al. 2011). However, the present result contributes new knowledge with the finding that sickness presenteeism is also associated with suicidal ideation.

Among Swedish surgeons, the multivariate model indicates that regular meetings to discuss stressful situations at work could be protective against suicidal ideation. These meetings may offer social support and thereby contribute to a better work environment and reduce the risk of suicidal ideation (Fridner et al. 2011; Wada et al. 2011). Additionally, the meetings could provide clearer information about roles and work situations (Robstad Andersen et al. 2010).

### Limitations

This study is based on cross-sectional data, and causal interpretations are therefore limited. A potential source of bias could be from self-report on outcome variable and work stressors and behaviours within the same questionnaire. We attempted to counteract this by not explicitly stating that having suicidal thoughts was being assessed as an outcome variable, as the survey covered a wide area including work environment and job assignments, as well as mental health.

Since only actively working surgeons were eligible, it is possible that the selection only included those who were relatively healthy, which could lead to an underestimation of suicidal ideation. Response rate for the surgeons in specific is unfortunately unfeasible to measure because some were employed at other sections than surgery, but still answered the survey as a surgical specialist. This is important to take into consideration when generalizing our results.

When comparing suicide ideation among the general public in Sweden and Italy, only lifetime prevalence were able to find. This may be important to acknowledge when comparing data.

## Conclusions

Contributing factors to suicidal ideation among surgeons are found to be work-related, and one of the most important is harassment. Depression was not found to be associated with suicidal ideation. Social support, such as regular meetings to discuss stressful situations at work, was found to have a protective function against suicidal ideation. It is important to spread this knowledge to management and HR, so that they can implement good strategies and offer better institutional support to their employees.
